# Enhanced exclusive enteral nutrition delivery during the first 7 days is associated with decreased 28-day mortality in critically ill patients with normal lactate level: a post hoc analysis of a multicenter randomized trial

**DOI:** 10.1186/s13054-024-04813-6

**Published:** 2024-01-20

**Authors:** Yizhe Chen, Zirui Liu, Qiuhui Wang, Fei Gao, Hongyang Xu, Lu Ke, Zheng-Yii Lee, Christian Stoppe, Daren K. Heyland, Fengming Liang, Jiajia Lin, for the Chinese Critical Care Nutrition Trials Group (CCCNTG)

**Affiliations:** 1 Department of Critical Care Medicine, The Affiliated Wuxi People’s Hospital of Nanjing Medical University, Wuxi Medical Center, Nanjing Medical University, No. 299 Qingyang Road, Wuxi, 214043 China; 2 Department of Critical Care Medicine, Jinling Hospital, Affiliated Hospital of Medical School, Nanjing University, No. 305 East Zhongshan Road, Nanjing, 210002 China; 3 Research Institute of Critical Care Medicine and Emergency Rescue at Nanjing University, Nanjing, China; 4 Department of Anaesthesiology, Faculty of Medicine, University of Malaya, Kuala Lumpur, Malaysia; 5 Department of Cardiac Anesthesiology and Intensive Care Medicine, Charité, Berlin, Germany; 6 Department of Anaesthesiology, Intensive Care, Emergency and Pain Medicine, University Hospital Würzburg, Würzburg, Germany; 7 Department of Critical Care Medicine, Queen’s University, Angada 4, Kingston, ON K7L 2V7 Canada; 8 Clinical Evaluation Research Unit, Kingston General Hospital, Angada 4, Kingston, ON K7L 2V7 Canada; 9 Chinese Critical Care Nutrition Trials Group (CCCNTG), No. 22 Hankou Road, Nanjing, 210093 China

**Keywords:** Enteral nutrition, Lactate, Mortality, Critical illness, Nutritional support

## Abstract

**Background and aims:**

Exclusive enteral nutrition (EN) is often observed during the first week of ICU admission because of the extra costs and safety considerations for early parenteral nutrition. This study aimed to assess the association between nutrition intake and 28-day mortality in critically ill patients receiving exclusive EN.

**Methods:**

This is a post hoc analysis of a cluster-randomized clinical trial that assesses the effect of implementing a feeding protocol on mortality in critically ill patients. Patients who stayed in the ICUs for at least 7 days and received exclusive EN were included in this analysis. Multivariable Cox hazard regression models and restricted cubic spline models were used to assess the relationship between the different doses of EN delivery and 28-day mortality. Subgroups with varying lactate levels at enrollment were additionally analyzed to address the potential confounding effect brought in by the presence of shock-related hypoperfusion.

**Results:**

Overall, 1322 patients were included in the analysis. The median (interquartile range) daily energy and protein delivery during the first week of enrollment were 14.6 (10.3–19.6) kcal/kg and 0.6 (0.4–0.8) g/kg, respectively. An increase of 5 kcal/kg energy delivery was associated with a significant reduction (approximately 14%) in 28-day mortality (adjusted hazard ratio [HR] = 0.865, 95% confidence interval [CI]: 0.768–0.974, *P* = 0.016). For protein intake, a 0.2 g/kg increase was associated with a similar mortality reduction with an adjusted HR of 0.868 (95% CI 0.770–0.979). However, the benefits associated with enhanced nutrition delivery could be observed in patients with lactate concentration ≤ 2 mmol/L (adjusted HR = 0.804 (95% CI 0.674–0.960) for energy delivery and adjusted HR = 0.804 (95% CI 0.672–0.962) for protein delivery, respectively), but not in those > 2 mmol/L.

**Conclusions:**

During the first week of critical illness, enhanced nutrition delivery is associated with reduced mortality in critically ill patients receiving exclusive EN, only for those with lactate concentration ≤ 2 mmol/L.

*Trial registration*: ISRCTN12233792, registered on November 24, 2017.

**Supplementary Information:**

The online version contains supplementary material available at 10.1186/s13054-024-04813-6.

## Introduction

Enteral nutrition (EN) provides unique non-nutritional benefits, including maintenance of gastrointestinal integrity, preservation of intestinal microbiome, and modulation of the immune and inflammatory responses [1–4]. Several studies have shown that early EN may improve outcomes in critically ill patients [5–8]. Therefore, the current guidelines strongly recommend initiating EN within 48 h after ICU admission if there is no contraindication [9, 10], and supplemental parenteral nutrition (PN) remains controversial within the first week [9]. As a result, exclusive EN delivery becomes a common practice during the first week of ICU admission. However, the progression of EN into a target-reaching dose is highly subjective to the clinician and often takes several days due to feeding intolerance or other adverse events [11–13].

Although early EN has become the standard of care in critically ill patients [9, 10], a major concern impeding early EN is unstable hemodynamics. The guidelines recommended EN be withheld until the patient is fully resuscitated and/or hemodynamically stable [9], and serum lactate is a widely used marker for hypoperfusion in shock patients [14–17]. Also, there is a concern that too much EN delivery may cause mesenteric ischemia in patients with insufficient gastrointestinal perfusion, evidenced by increased lactate [18, 19]. Nevertheless, there is a lack of evidence regarding whether patients with or without increased blood lactate may respond differently to enhance EN delivery.

In this study, we aimed to evaluate the association between energy and protein delivery and 28-day mortality in critically ill patients receiving exclusive EN during the first week of stay. Furthermore, we also stratified the study subjects according to their blood lactate level at enrollment to assess potential interaction.

## Methods

### Study design and patients

This was a post hoc analysis of data from a multicenter, cluster-randomized controlled trial (NEED trial) [20]. The trial was approved by the ethics committee of Jinling Hospital (22017NZKY-019–02) and registered at the ISRCTN registry (ISRCTN12233792) before enrollment. Written informed consent was obtained from all patients or next of kin. Additional information on the NEED trial, including the study protocol and statistical analysis plan, was published in the main article [20].

Overall, 2772 newly admitted patients were enrolled from 90 ICUs across China. Briefly, the participating ICUs were randomized in a 1:1 ratio to either implementing a feeding guideline or following a routine practice. In the guideline group, a nutrition support team was formed to actively implement the guideline using a graphical feeding protocol, instructing when to initiate EN, when to adjust the feeding rate, when to consider parenteral nutrition, and how to manage intolerance. Moreover, daily checklists, standardized educational materials, and live online education outreach meetings were used to facilitate the implementation of the feeding guideline. Meanwhile, other ICUs in the control group followed the local clinical practice and remained unaware of the guideline content. The original study was partly funded by Nutricia, Wuxi, China, which had no role in the study's design, data collection, analysis, or preparation of the manuscript. Representatives from Nutricia received copies of the paper before formal submission but had no influence over the content.

In this post hoc analysis, patients who stayed in the ICU for at least 7 days and received exclusive EN during the first week of enrollment were included. We excluded the patients who received any oral diet because the energy and protein via oral intake cannot be accurately calculated.

### Data collection

All the data required for this analysis were collected from the electronic database of the original trial, including baseline characteristics, daily nutritional therapy, and the requirement of organ support therapy. The baseline characteristics were collected if patients admitted to the participating ICUs were eligible for inclusion. The baseline data included age, sex, height, body mass index (BMI), types of ICU admission, number of co-morbidities, illness severity scores such as Sequential Organ Failure Assessment (SOFA) score [21], Acute Physiology and Chronic Health Evaluation II (APACHE II) score [22], and modified Nutrition Risk in the Critically ill (mNUTRIC) score [23], and the most recent lactate level at enrollment. Daily information on nutrition therapy, including the time to initiation of EN, the amount of energy and protein delivered by EN, and the use of prokinetic agents, were collected for a maximum of 7 days after enrollment or until ICU discharge or death. The use of organ support therapy (renal replacement therapy, mechanical ventilation, and vasoactive agents) was collected during the same time period.

### Outcomes and definition

The primary outcome is 28-day mortality. The secondary outcome is ICU-free days to day 28, which is defined as days alive and free from the need for intensive care from enrollment to day 28. Patients who were discharged from ICU on day 28 or died prior to day 28 were assigned zero ICU-free days. Nutrition adequacy was defined as the actual daily energy or protein delivered in the first 7 days divided by the target nutritional requirement as a percentage. The nutrition targets were defined according to the original trial [20], which were 25 kcal/kg of the ideal body weight (IBW) for energy delivery and 1.2 g/kg (IBW) for protein delivery. The IBW was calculated using the Broca formula: Height (cm) − 100 (men)/105 (women) [24].

### Statistical analyses

The Kolmogorov–Smirnov test was used to examine the normality of continuous variables. Continuous data were presented in mean and standard deviation or median and interquartile range (IQR). Categorical data were presented as frequencies and percentages.

The Cox proportional hazards models were performed to assess the association between nutrition delivery and 28-day mortality. Energy and protein were modeled separately due to their high co-linearity. Potential confounders, including age, sex, BMI, SOFA score, number of co-morbidities, and the study interventions (guideline group or control group) (Additional file 1: Table S1), were additionally adjusted in the models. Hazard ratio (HR) and 95% confidence interval (CI) were estimated per increased 5 kcal/kg of energy or 0.2 g/kg of protein, respectively. Additionally, we performed subgroup analyses according to the blood lactate levels at enrollment (> 2 mmol/L and ≤ 2 mmol/L) [25]. Restricted cubic spline models with random intercepts were fitted to explore the dose–response relationship of nutrition delivery (energy and protein delivery in separate models) and 28-day mortality. The adjusted factors were the same as those in the Cox proportional hazards models. To address the potential impact of using IBW instead of actual body weight (ABW), we performed a sensitivity analysis to support the primary analysis. In this analysis, we used the ABW to calculate the daily energy (kcal/kg/d) and protein (g/kg/d) in the Cox proportional hazards models.

All statistical analyses were performed using R software (version 4.1.0). A two-tailed *P *value of < 0.05 was considered significant.

## Results

### Patients characteristics

**Fig. 1:**
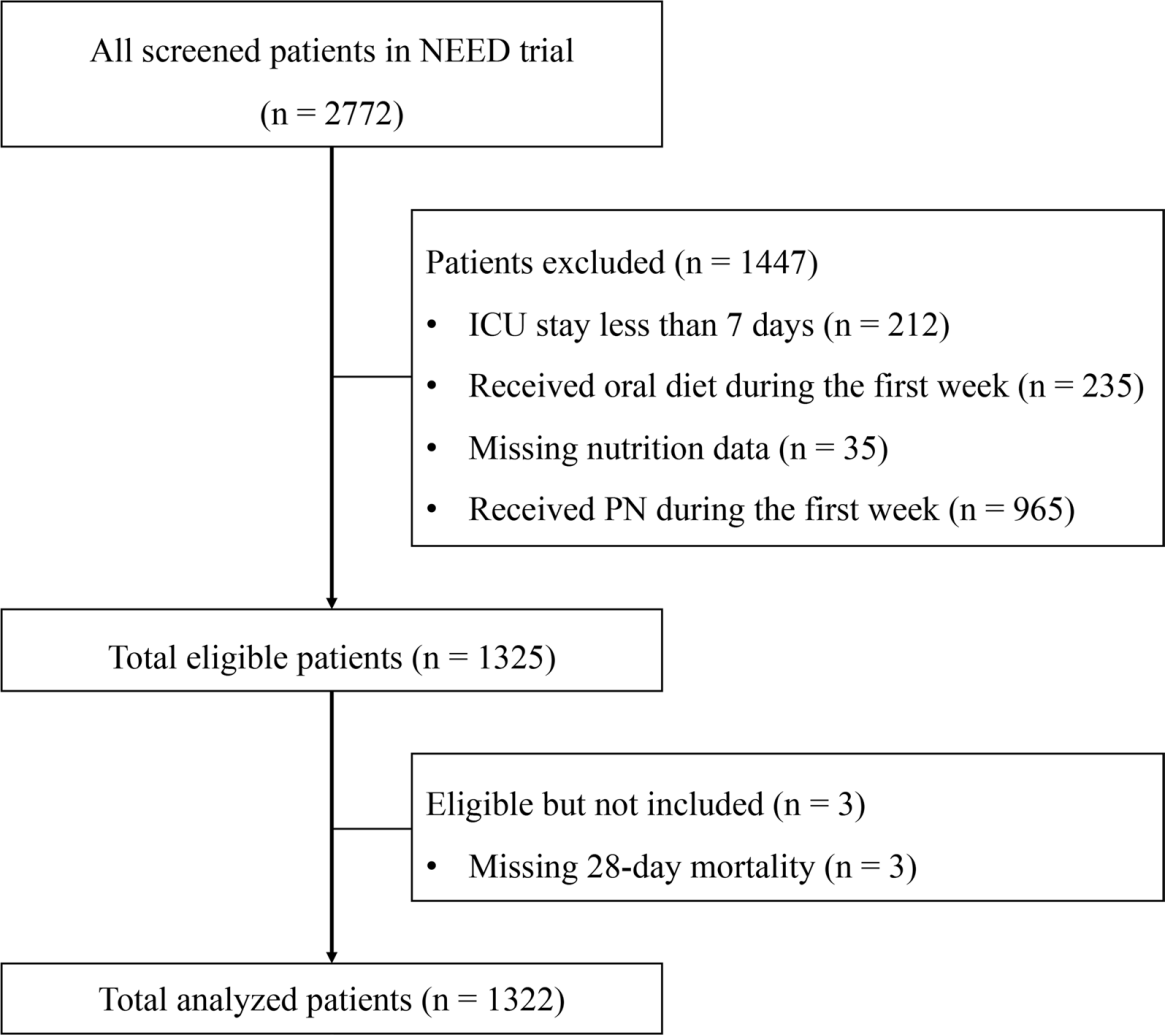
Flowchart of study patients

After screening all patients in the NEED trial, 1322 patients were included in this analysis (Fig. 1). Table 1 describes the baseline characteristics and clinical outcomes of the study patients. Overall, 66% of the study subjects were male, with a median age of 64 (IQR 49–76) years and a median SOFA score of 7 (IQR 5–10). The majority of the study subjects were admitted to general ICUs (*n* = 1143, 86.5%), underwent nutrition therapy with a feeding protocol (*n* = 822, 62.2%), and required mechanical ventilation (*n* = 844, 63.8%). About 4% of patients were admitted to surgical ICUs, and 1.6% of patients were admitted to medical ICUs, respectively. The 28-day mortality of the study cohort was 13.2% (175/1322).

**Table 1:** Baseline characteristics and clinical outcomes of study patients

	Total *n* = 1322
Age, y	64 (49–76)
BMI, kg/m^2^	22.5 (20.8–24.5)
Male	873 (66)
APACHE II	18 (14–23)
SOFA	7 (5–10)
mNUTRIC score	4 (3–6)
Number of co-morbidities	2 (1–3)
Lactate, mmol/L	1.8 (1.2–3)
ICU admission type	
General	1143 (86.5)
Emergency	105 (7.9)
Surgical	53 (4)
Medical	21 (1.6)
Study intervention	
Guideline group	822 (62.2)
Control group	500 (37.8)
Organ support therapy at enrollment	
CRRT	141 (10.7)
Mechanical ventilation	844 (63.8)
Vasoactive agents	398 (30.1)
Clinical outcome	
28-day mortality	175 (13.2)
ICU-free day to 28 days	6 (0–18)

Data are presented as n (%) or median (interquartile range)

BMI, Body Mass Index; APACHE II, Acute Physiology and Chronic Health Evaluation II; SOFA, Sequential Organ Failure Assessment; mNUTRIC, modified Nutrition Risk in the Critically ill; and CRRT, Continuous Renal Replacement Therapy

### Nutrition therapy

The information on enteral nutrition therapy is summarized in Table 2. Most patients (*n* = 1004, 75.9%) received EN within 48 h after ICU admission, and the median time to start EN was 2 (IQR 1–2) days. About 21.1% of patients received prokinetic agents during the first week. Gastric feeding was the predominant route for initiation of EN (91.6% of patients). On average, the study patients received 14.6 kcal/kg/d for energy delivery and 0.6 g/kg/d for protein delivery during the first week after enrollment, accounting for 58.5% adequacy of energy delivery and 44% adequacy of protein delivery, respectively. The daily energy and protein delivery within the first 7 days after enrollment are shown in Additional file 1: Figure S1.

**Table 2:** Nutrition therapy of study patients

	Total *n* = 1322
Nutrition process during the first week	
Time to start EN, day	2 (1–2)
Patients receiving EN within 48 h after ICU admission	1004 (75.9)
Patients receiving prokinetic agents	279 (21.1)
Route to start EN	
Gastric feeding	1211 (91.6)
Post-pyloric feeding	111 (8.4)
Mean energy and protein delivery after enrollment during the first week	
Energy delivery, kcal/day	928.6 (654.3–1214.3)
Energy delivery, kcal/kg/day	14.6 (10.3–19.6)
Adequacy of energy delivery, %	58.5 (41.3–78.3)
Protein delivery, g/day	36.7 (25.7–48)
Protein delivery, g/kg/day	0.6 (0.4–0.8)
Adequacy of protein delivery, %	44 (31.2–59)

Data are presented as n (%) or median (interquartile range)

EN, enteral nutrition and ICU, intensive care unit

### Association between enhanced nutrition delivery and clinical outcomes

The relationship between enteral nutrition delivery and 28-day mortality is shown in Table 3. During the first week of enrollment, each 5 kcal/kg increase in mean energy delivery was associated with an approximately 13% reduction in 28-day mortality (adjusted HR = 0.865, 95% CI 0.768–0.974, *P* = 0.016), while each 0.2 g/kg increase in mean protein intake (adjusted HR = 0.884, 95% CI 0.804–0.971, *P* = 0.01) was also associated with similarly reduced mortality.

**Table 3:** The relationship between enteral nutrition and 28-day mortality

	Unadjusted			Adjusted^*^		
	Hazard ratio	95% CI	P value	Hazard ratio	95% CI	P value
Total study population (*n* = 1332)						
Energy delivery (per 5 kcal/kg)	0.843	0.755–0.941	0.002	0.865	0.768–0.974	0.016
Protein delivery (per 0.2 g/kg)	0.849	0.759–0.949	0.004	0.868	0.770–0.979	0.021
*Subgroup analysis*						
Lactate concentration ≤ 2 mmol/L (*n* = 774)						
Energy delivery (per 5 kcal/kg)	0.795	0.675–0.935	0.006	0.804	0.674–0.960	0.016
Protein delivery (per 0.2 g/kg)	0.801	0.679–0.946	0.009	0.804	0.672–0.962	0.017
Lactate concentration > 2 mmol/L (*n* = 548)						
Energy delivery (per 5 kcal/kg)	0.915	0.788–1.063	0.247	0.933	0.796–1.095	0.396
Protein delivery (per 0.2 g/kg)	0.921	0.791–1.072	0.288	0.941	0.800–1.105	0.457

^*^Adjusted for age, sex, BMI, study interventions, SOFA, and number of co-morbidities

**Fig. 2:**
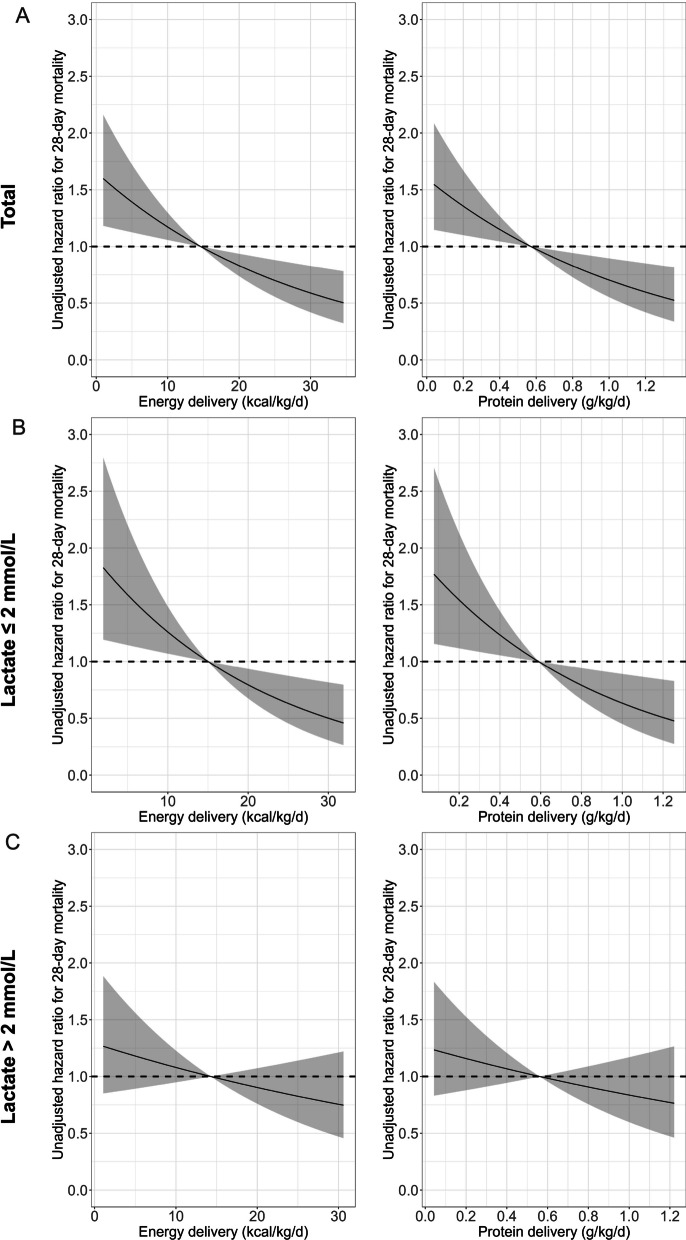
Association of energy (kcal/kg/d) with 28-day mortality (left), and protein (g/kg/d) delivery with 28-day mortality (right) in total population (**A**), patients with lactate ≤ 2 mmol/L (**B**) or > 2 mmol/L (**C**)

In subgroup analysis, the association between enhanced nutrition delivery and improved 28-day survival remained significant in patients with baseline lactate concentration ≤ 2 mmol/L (adjusted HR = 0.804, 95% CI 0.674–0.960 for energy delivery and adjusted HR = 0.804, 95% CI 0.672–0.962 for protein delivery, respectively), but not for patients with a lactate level over 2 mmol/L (adjusted HR = 0.933, 95% CI 0.796–1.095 for energy delivery and adjusted HR = 0.941, 95% CI 0.800–1.105 for protein delivery, respectively). The restricted cubic spline analysis results were consistent with the primary analysis (Fig. 2), including the subgroup analysis.

Considering the potential impact of using IBW instead of ABW, we performed a sensitivity analysis using the ABW to calculate the daily energy (kcal/kg/d) and protein (g/kg/d). The results remained stable in different models (Additional file 1: Table S2).

## Discussion

In this study, the results showed that enhanced nutrition delivery was associated with reduced 28-day mortality in critically ill patients receiving exclusive EN during the first week of ICU admission. However, this association disappeared in patients with baseline lactate concentration > 2 mmol/L.

Our findings demonstrated that the nutrition therapy was, on average, suboptimal in our study, with only 58.5% adequacy for energy delivery and 44% adequacy for protein delivery during the first week, respectively. However, these findings align with comparable observational studies, which showed that most ICU patients receive only approximately 50% of their energy and protein requirements [26], suggesting a significant gap between guideline recommendations and clinical practice. Moreover, exclusive EN feeding may lead to nutritional inadequacy due to implementation factors such as feeding intolerance [27], feeding interruption [28], unstable hemodynamics [29], etc., although the clinical significance was not well demonstrated. Supplemental PN might be a promising option to close these gaps, but there are concerns about the optimal timing of SPN and the risk of overfeeding without the guidance of indirect calorimetry [30].

Studies focusing on patients receiving exclusive EN are scarce in the literature. Two observational studies involving exclusively EN-fed septic patients found that more EN delivery was associated with reduced 60-day mortality, more ventilator-free days in septic patients, and fewer infectious complications [11, 12]. However, two randomized trials comparing trophic EN and full EN feeding for the first 6 days of randomization in acute respiratory failure/acute lung injury patients did not detect a significant difference in clinical outcomes [31, 32]. Moreover, in two previous large trials comparing different energy delivery strategies (the TARGET [33] and the PermiT [34]), the proportions of PN-fed patients were both lower than 5%, making the study populations very similar to exclusive EN-fed patients. However, either enhanced energy delivery (the TARGET) or permissive underfeeding (the PERMIT) did not improve mortality. Compared to the abovementioned studies, our study excluded those with a short ICU stay (less than 7 days), in whom nutrition therapy is less likely to affect outcomes. Still, the results need to be confirmed in a future trial.

For shock patients, the 2016 Society of Critical Care Medicine (SCCM) and American Society for Parenteral and Enteral Nutrition (ASPEN) guidelines recommend delaying EN until the patient is fully resuscitated and/or hemodynamically stable [9]. Lactate level was found to be significantly correlated with microcirculation perfusion in shock patients [35], and serum lactate concentration > 2 mmol/L is accepted as an indicator of septic shock according to Sepsis 3.0 [36]. Our subgroup analysis indicated that the beneficial effect of EN was more evident in patients with lactate concentration ≤ 2 mmol/L, whereas patients with lactate concentration > 2 mmol/L may not benefit from enhanced EN delivery. One possible explanation is that high lactate levels might be associated with intestinal hypoperfusion and an increased risk of feeding intolerance [37], which hampers the benefits of enhanced EN delivery. Another possible explanation is that the initiation of EN was delayed in the high lactate group, as required by the feeding guideline in the original trial (not to start EN when lactate level > 4.0 mmol/L). Due to delayed EN initiation, these patients may need more EN-fed time to benefit from enhanced EN delivery, and our observation window (7 days) limited our ability to test this possibility. Future studies may extend the observation time to assess the effect of nutrition therapy in this population.

We acknowledge several limitations of this study. First, owing to the post hoc nature of this study, a causal relationship between higher EN delivery and improved 28-day survival cannot be inferred. Second, the Chinese critically ill population is significantly different from the American or European population in terms of the proportion of obese patients [38, 39], which may impact the generalizability of our findings. Third, we did not collect the adverse events of enteral feeding, such as acute mesenteric ischemia. Additionally, due to the multicollinearity between energy and protein delivery, we could not weigh which factor is more important.

## Conclusion

This study showed that a greater amount of EN delivery is associated with decreased 28-day mortality in critically ill patients, only in patients with lactate concentration ≤ 2 mmol/L. Further prospective studies are warranted to confirm our findings.

### Supplementary Information

**Additional file 1.** **Table S1**. Univariable Cox analysis for 28-day mortality. **Table S2**. Sensitivity analysis for the relationship between enteral nutrition and 28-day mortality. **Figure S1**. Daily enteral nutrition delivery.

## Data Availability

The datasets generated and analyzed in this article are not publicly available due to health privacy concerns but are available from the corresponding author on reasonable request.

## Abbreviations

ICU: Intensive care unit
EN: Enteral nutrition
BMI: Body mass index
mNUTRIC: Modified nutrition risk in the critically ill
APACHE II: Acute physiology and chronic health evaluation II
SOFA: Sequential organ failure assessment
CRRT: Continuous renal replacement therapy
MV: Mechanical ventilation
