# Supplementary material for: Enhanced exclusive enteral nutrition delivery during the first 7 days is associated with decreased 28-day mortality in critically ill patients with normal lactate level: a post hoc analysis of a multicenter randomized trial
Source: Crit Care. 2024 Jan 20;28:26. doi: 10.1186/s13054-024-04813-6 (PMC10799529; doi:10.1186/s13054-024-04813-6)
Supplement: Supplementary file 1 — Additional file 1. Table S1. Univariable Cox analysis for 28-day mortality. Table S2. Sensitivity analysis for the relationship between enteral nutrition and 28-day mortality. Figure S1. Daily enteral nutrition delivery. [file 13054_2024_4813_MOESM1_ESM.docx]

Supplementary Table 1. Univariable Cox analysis for 28-day mortality

|  | Hazard ratio | 95% CI | P value |
| --- | --- | --- | --- |
| Age | 1.009 | 1-1.017 | 0.043 |
| Male | 0.965 | 0.706-1.318 | 0.821 |
| BMI | 0.904 | 0.861-0.95 | <0.001 |
| APACHE II | 1.058 | 1.036-1.08 | <0.001 |
| SOFA | 1.082 | 1.039-1.126 | <0.001 |
| mNUTRIC score | 1.204 | 1.117-1.298 | <0.001 |
| Number of co-morbidities | 1.097 | 1.024-1.175 | 0.008 |
| Study interventions | 1.184 | 0.871-1.611 | 0.282 |

BMI, Body Mass Index; APACHE II, Acute Physiology and Chronic Health Evaluation II; SOFA, Sequential Organ Failure Assessment; mNUTRIC, modified Nutrition Risk in the Critically ill.

Supplementary Table 2. Sensitivity analysis for the relationship between enteral nutrition and 28-day mortality

|  | Unadjusted | | |  | Adjusted^*^ | | |
| --- | --- | --- | --- | --- | --- | --- | --- |
|  | Hazard ratio | 95% CI | P value |  | Hazard ratio | 95% CI | P value |
| **Total study population (n = 1332)** |  |  |  |  |  |  |  |
| Energy delivery (per 5 kcal/kg) | 0.900 | 0.811-0.998 | 0.046 |  | 0.866 | 0.775-0.969 | 0.012 |
| Protein delivery (per 0.2 g/kg) | 0.905 | 0.814-1.005 | 0.062 |  | 0.866 | 0.774-0.970 | 0.013 |
| **Subgroup analysis** |  |  |  |  |  |  |  |
| **lactate concentration ≤ 2 mmol/L (n = 774)** |  |  |  |  |  |  |  |
| Energy delivery (per 5 kcal/kg) | 0.891 | 0.768-1.034 | 0.128 |  | 0.824 | 0.700-0.969 | 0.02 |
| Protein delivery (per 0.2 g/kg) | 0.897 | 0.772-1.043 | 0.159 |  | 0.821 | 0.696-0.969 | 0.019 |
| **lactate concentration > 2 mmol/L (n = 548)** |  |  |  |  |  |  |  |
| Energy delivery (per 5 kcal/kg) | 0.935 | 0.808-1.083 | 0.371 |  | 0.924 | 0.789-1.081 | 0.323 |
| Protein delivery (per 0.2 g/kg) | 0.940 | 0.810-1.091 | 0.414 |  | 0.919 | 0.787-1.073 | 0.286 |

^*^ Adjusted for age, sex, BMI, study interventions, SOFA and number of co-morbidities.

Supplementary Figure 1. Daily enteral nutrition delivery


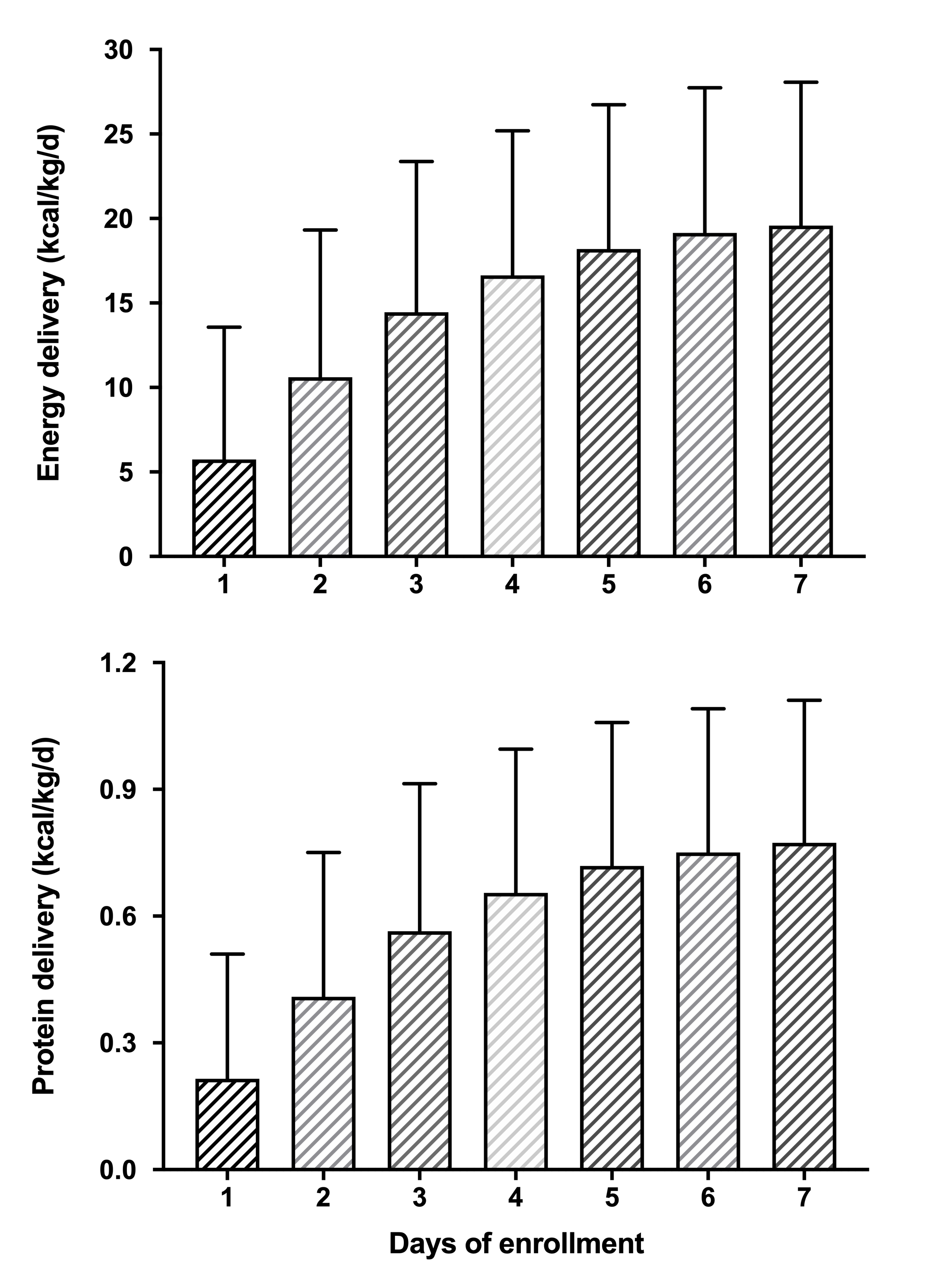


Error bars indicate the standard deviation.
